# Emerging roles of cannabinoid pathways in renal injury and repair

**DOI:** 10.3389/fphar.2026.1777644

**Published:** 2026-07-27

**Authors:** Ricardo Romero-Guevara, Sergio Guzmán-Rodríguez, Bruno A. Marichal-Cancino

**Affiliations:** Department of Physiology and Pharmacology, Centre of Basic Sciences, Autonomous University of Aguascalientes, Ciudad Universitaria, Aguascalientes, Mexico

**Keywords:** acute kidney injury (AKI), cannabinoids, CB1, CB2, cannabinoid receptors, chronic kidney disease, GPR55

## Abstract

Chronic kidney disease (CKD) is a significant health problem around the world. It can progress towards end-stage renal disease (ESRD), in which the current therapeutic options are dialysis and kidney transplant, which have several challenges in therapy compliance and finding a donor. There is a crosstalk between high blood pressure, diabetes, and obesity, which are the leading causes of CKD. The increased prevalence of these conditions, plus the ageing population, highlights the importance of finding new therapies to prevent and stop the progression of CKD. At the cellular level, CKD is characterized by the progressive loss of podocytes in the kidney glomeruli, reducing their filtration capacity. Other hallmarks of CKD are also part of acute kidney injury (AKI) pathogenesis, and both conditions are interlinked. The renal system expresses cannabinoid receptor types 1 and 2 (CB1 and CB2, respectively). There is evidence in animal models that targeting the cannabinoid receptors could modulate the progression of CKD and AKI. The role of cannabinoid signalling has not been sufficiently explored; several receptors that are not fully characterized have emerged as putative cannabinoid receptors (i.e., GPR55, GPR18, and GPR119), and some have been implicated in kidney pathophysiology. Moreover, cannabinoid receptors also interact and/or regulate other receptors (e.g., AT1, TRPV1, and PPARγ). Thus, there are several open questions regarding the role that cannabinoid signalling may play in the context of kidney diseases. In this mechanistic review, we examined the pharmacological actions of classic and emerging cannabinoid receptors on the renal system to identify potential therapeutic targets for kidney disease.

## 1 Introduction

### 1.1 Chronic kidney disease (CKD): an unmet medical need

CKD is a rising global health issue affecting approximately 9.1% of the population (approximately 850 million people) (GBD Chronic Kidney Disease Collaboration, 2020). The death rate of this pathology increased significantly in the last 2 decades and, according to projections, it is likely to increase substantially in the following years (Kovesdy, 2022). Diseases such as diabetes, obesity, and high blood pressure, which have all increased in recent decades, are independent risk factors for CKD and often coexist. For instance, diabetic nephropathy (DN) represents one of the most clinically significant complications of diabetes and a leading cause of CKD. A measure of morbidity and mortality, known as disability-adjusted life years (DALYs), indicates that 30% of DALYs in CKD are attributable to diabetes, while high blood pressure accounts for another third of cases of CKD (Kramer et al., 2019; Deng et al., 2025). Moreover, population ageing has also contributed to the increase in CKD prevalence. Therefore, more research is needed to investigate potential therapeutics to interrupt or delay the progression of this increasing and potentially lethal health problem.

Death from cardiovascular events and progress towards end-stage renal disease (ESRD) are critical outcomes of CKD; patients with ESRD can only be treated by dialysis and/or kidney transplant. However, these therapies are insufficient and often hamper the quality of life of patients. For example, patients in dialysis have a higher risk of infection and premature death (Mailloux et al., 1991; Kramer et al., 2019). In fact, a pan-European study showed that dialysis is associated with a critical reduction in life expectancy, often to nearly one-third of that of the general population (Kramer et al., 2019). On the other hand, while the life expectancy of transplanted kidney patients is better than those on dialysis, it is still 30% less than that of the age-matched population (Kramer et al., 2019). In addition, there is a shortage of donors for kidney transplants, according to the US government, of almost 90,000 patients added each year to the kidney transplant waiting list, only 27,000 are transplanted (Organ Donation Statistics, 2025); moreover, patients who need transplantation require a lifetime immunosuppression regimen.

In addition to the impact on patients health, the burden of ESRD on healthcare systems is also significant. For example, 2%–4% of the world’s healthcare budget is allocated to dialysis and kidney transplants even though they represent 0.15% of the total patient population (Vanholder et al., 2017; Jommi et al., 2018; Li et al., 2021). In a study carried out in Turkey, the cost of ESRD (kidney transplantation and dialysis) contributed to 5.5% of the national healthcare budget (Erek et al., 2004). In the same line, a study conducted on the NHS (United Kingdom) showed that ESRD, representing 2% of CKD patients, needs more than half of the budget allocated for CKD care (800 million USD approximately) (Kerr et al., 2012). Indeed, worldwide, the yearly cost of dialysis increases approximately 20-fold compared to the healthcare cost of earlier stages of CKD (106 824 vs. 5,168 USD per patient), similarly, the cost of kidney transplant and treatment the following year is much higher than the healthcare cost of earlier CKD stages (91 988 vs. 5,168 USD per patient) (Jha et al., 2023). For these reasons, in some countries, dialysis is insufficiently available to all patients who need it. In fact, it is estimated that more than 2.2 million people worldwide died prematurely due to the lack of access to dialysis treatment (Liyanage et al., 2015). Largely, ESRD comprises an economic burden that challenges the sustainability, accessibility, and equity of healthcare systems worldwide.

### 1.2 The pharmaceutical landscape for CKD patients

The renin-angiotensin-aldosterone system (RAAS) is over-activated in diabetic kidney disease and other nephropathies, and there is compelling evidence that angiotensin receptor blockers (ARB) can be beneficial for many CKD patients (Leehey et al., 2000). In models of nephrotic syndrome, angiotensinogen and angiotensin receptors are increased in glomeruli, while ARB administration counteracts this effect (Yamazaki et al., 2016). Inhibitors of RAAS are the most widely used pharmaceuticals for the treatment of CKD patients. These drugs have shown effectiveness beyond their hemodynamic effects. For example, it has been observed in diabetic (Björck et al., 1992) and non-diabetic patients (The Lancet, 1997) that in the groups treated with RAAS inhibitors, a decrease in proteinuria was observed compared to other antihypertensive drugs (β blockers) despite similar reduction in blood pressure with both medications. Nonetheless, these drugs are ineffective in many patients and, at most, diminish the rate of CKD progression. Although the RAAS function in the kidney is incompletely characterized, it is well established that there is an intrarenal upregulation of RAAS in diabetes models (Anderson, Jung and Ingelfinger, 1993; Vallon and Komers, 2011), with several consequences such as excess glucose intake in podocytes (Lewko et al., 2018) and its associated toxicity (Vallon and Komers, 2011). Glucose toxicity is also involved in tubular pathophysiology in diabetic patients, explaining why the sodium-glucose cotransporter 2 (SGLT2) inhibitors have shown promise in the management of DN (Wanner et al., 2016; Perkovic et al., 2019). Along this line, evidence from CKD models suggests that targeting the cannabinoid system can modulate nutrient sensing in the kidney. Moreover, recreational cannabinoid use may be linked to potential harmful effects for the kidneys (Srisung, Jamal and Prabhakar, 2015; Rein and Wyatt, 2018). However, there are no current approved therapies involving synthetic cannabinoids for CKD patients, and therefore, more research is needed before moving to the clinic.

### 1.3 The pathophysiology of CKD and acute kidney injury (AKI)

CKD is characterized by progressive glomerular and tubular damage. The glomerulus is the filtration barrier of the kidney, made of capillary tufts composed of fenestrated endothelial cells, the glomerular basement membrane, and specialized epithelial cells called podocytes. At the cellular level, the progression of CKD is characterized by lessening of the podocyte basement membrane, loss of podocyte slit diaphragm proteins (e.g., nephrin and podocin), effacement of foot processes, cell death, mesangial expansion, tubule collapse, and interstitial tubular fibrosis (Nagata, 2016). Even though glomerular damage often occurs first in CKD, tubular damage also contributes to disease progression, and tubulointerstitial fibrosis is considered a hallmark of the disease. At the clinical level, it is well established that AKI is a risk factor for the later development of CKD (Ishani et al., 2009; Chawla et al., 2011; Ferenbach and Bonventre, 2015), highlighting that incomplete tubular repair prompts kidney disease progression. Mechanistically, it is considered that excess filtration causes nutrient overload, increased energy demands, hypoxia, and interstitial fibrosis, among other mechanisms that take place in the kidney tubules (Schnaper, 2017; Hinden et al., 2022). Therefore, the pathophysiological roles of glomeruli and tubules are interconnected in the progression of CKD. Notably, cannabinoid receptors have been identified in several types of kidney cells, and their expression changes in response to kidney damage, highlighting the importance of studying this system as a potential therapeutic target (Park et al., 2017; Barutta et al., 2018). The specific localization of cannabinoid receptors within renal structures, such as glomeruli and tubules, and their physiological actions may reveal their pharmacological utility.

### 1.4 The endocannabinoid system (ECS) as a therapeutic target

The ECS is composed of the cannabinoid type 1 and 2 receptors (i.e., CB1 and CB2, respectively), their endogenous ligands, including anandamide (AEA) and 2-arachidonoylglycerol (2-AG), the enzymes involved in their synthesis/catabolism, and the corresponding genes. The ECS regulates physiological activities throughout the body, including thermal regulation, movement, blood pressure, pain perception, energy sensing, oxidative stress, immunity, inflammation, metabolism, cancer progression, and cell death, among others (Kogan and Mechoulam, 2007; Battista et al., 2012). The multiple actions of cannabinoids cannot be explained only by the classic receptors CB1/CB2, which are both coupled to G-i/o signalling pathways. Other targets of cannabinoids include peroxisome proliferator-activated receptors (PPARs) and TRPV1 channels. Also, the orphan GPR55 was proposed as the type three cannabinoid receptor (CB3) (Pertwee, 2007; Yang, Zhou and Lehmann, 2016; Kapur et al., 2009). These receptors are known to form heterodimers among themselves, exhibit promiscuity with their ligands, and modulate downstream effectors induced by other signalling systems, thereby adding to the complexity of the endocannabinoid signalling pathway (Kargl et al., 2012; Shore and Reggio, 2015). Along the same line, heteromers between cannabinoid receptors, dopamine, adenosine, opioid, and angiotensin receptors have also been reported (Rozenfeld et al., 2011, 2012; Sierra et al., 2019; Rivas-Santisteban et al., 2023; Mińczuk et al ., 2022). The latter one is known to play a role in kidney disease progression. A physical interaction and mutual regulation of cannabinoid and angiotensin receptors in the kidney remains to be clarified, but CB1 and RAAS activation are both upregulated in CKD models. Interestingly, there is evidence in the liver that both receptors (CB1 and AT1) interact with each other, in which CB1 potentiates the profibrotic effects of AT1 in hepatic stellate cells of rats exposed to long-term ethanol consumption (Rozenfeld et al., 2011). Notably, in the kidney AT1 activation induces TGF-β, fibrosis in mesangial cell cultures, as well as with angiotensin II administration *in vivo* (Kagami et al., 1994). Therefore, it is necessary to investigate if fibrosis by RAAS activation is mediated by endocannabinoids and the mechanisms of their regulation.

Only a few clinically approved cannabinoid medicines are available on the market. Most of them reach neurons in the central nervous system, and their uses include chronic pain, anorexia, epilepsy, and neurodegenerative disorders (Kogan and Mechoulam, 2007; Bilbao and Spanagel, 2022). Nonetheless, at the preclinical stage, the potential application of cannabinoids is even more promising. For instance, cannabinoid receptors have been found in many organs and cell types, such as the liver, kidney, heart, skeletal muscle, adipocytes, pancreatic cells, endothelial cells, and immune cells, and at high abundance in the CNS and peripheral neurons (Haspula and Clark, 2020). Regrettably, the functions of cannabinoids in many organs are complex and remain partially understood. The above difficulties affect the use of this system for therapeutic actions. In this review, we revisit the pathway’s complexity, conflicting evidence, and the therapeutic potential of targeting the ECS for the treatment of kidney diseases.

## 2 The ECS dysregulation in kidney disease models

### 2.1 CB1 in glomerular pathophysiology and CKD

In DN and other kidney diseases, a dysregulation of the ECS has been reported in glomeruli and tubules in human and animal models (Barutta et al., 2018). For example, an upregulation of CB1 was observed in a mouse model of DN, and exposure to AM251 (a CB1 antagonist) improved albuminuria in streptozotocin-induced DN (Barutta et al., 2010). Along the same line, in another study, the expression and activity of CB1 in glomeruli were increased in a rat model of DN, while its blockade with JD5037 improved renal function, evidenced by reduced albuminuria and markers of inflammation, as well as improved glomerular filtration rate (Jourdan et al., 2014). These effects are thought to be mediated by a direct effect on podocytes, since the podocyte-specific deletion of CB1 improved the renal function in a mouse model of DN (Jourdan et al., 2018). Other authors have also seen improvements in albuminuria with AM251 treatment (Jenkin et al., 2015a). In line with these models, studies in human kidneys have also reported increased CB1 expression in glomeruli and tubules in patients with various nephropathies, including DN (Lecru et al., 2015). Hence, CB1 receptors may constitute interesting pharmacological targets for the treatment of renal injuries.

The ECS regulation differences could be explained by disease aetiology or species-specific singularities. For example, in human kidney samples from obese individuals who exhibited robust alterations in kidney function and structure, including the glomeruli, whole-kidney CB1 expression decreased compared to lean individuals (Permyakova et al., 2023). In contrast, in the obese Zucker rat, no difference in *Cnr1* mRNA expression was observed between obese (fa/fa) and lean rats (fa/+). This difference between human and rat CB1 expression in response to obesity may be explained by technical differences in sensitivity between western blot (Permyakova et al., 2023) and QPCR analysis (Janiak et al., 2007), a mismatch between protein and mRNA levels, or genetics since the Zucker fat rat is an inbreed strain with a mutation in the leptin receptor, which is known to alter several metabolic and hormonal axis, such as hyperleptinemia, which differ markedly to the pathophysiology of obesity in most people (Wang, Chandrasekera and Pippin, 2014). Nonetheless, in humans and animal models, there is a low level of expression of CB1 in the normal kidney that is upregulated in DN and other nephropathies (Janiak et al., 2007; Larrinaga et al., 2010; Lecru et al., 2015), while in obesity, it is still unclear how CB1 is regulated in the body. Despite these differences, the evidence so far suggests a positive role for CB1 antagonism in the treatment of CKD. For example, in the unilateral ureteral obstruction (UUO) mouse model, rimonabant, a CB1 antagonist decreased kidney fibrosis, a hallmark of kidney disease progression (Lecru et al., 2015), while in the Zucker obese rat model, Janiak and colleagues also showed reduced glomerular fibrosis, improved creatinine and urea clearance, and better metabolic profile (reduced glucose, triglycerides and cholesterol), upon rimonabant treatment (Janiak et al., 2007). Similarly, CB1 receptor blockade has been consistently associated with the preservation of glomerular structure and function (Barutta et al., 2010; Jourdan et al., 2014). This effect is evidenced by reduced albuminuria and decreased activation of the RAAS compared with Zucker diabetic fatty (ZDF) control rats (Jourdan et al., 2014). Mechanistically, CB1 activation has been shown to interfere with mitochondrial dynamics and ROS production by phosphorylation of dynamin 1 (Drori et al., 2019), and miRNA 29 has been shown to induce CB1 expression at expense PPARγ, leading to the expression of proinflammatory cytokines in a DN model (Tung et al., 2019). Collectively, these findings support the notion that CB1 receptor inactivation may represent a promising therapeutic strategy for the treatment of CKD.

Admittedly, CB1 antagonism decreases RAAS activation, blood pressure, glucose, body weight, and plasma triglycerides, which may contribute independently to kidney protection. Moreover, rimonabant and AM251 are non-selective antagonists of CB1, as they can also be GPR55 receptor antagonists and agonists, respectively (Ryberg et al., 2007). GPR55 has been recently implicated in kidney physiology (Simcocks et al., 2019; Chen et al., 2022). In fact, in the work of Simcocks and collaborators (Simcocks et al., 2019), the GPR55 agonist O1602 reduced body weight gain and leptin levels in a model of high-fat diet-induced obesity (DIO) rats. Therefore, it is possible that the improved metabolic profile observed in the DN study of Barutta (Barutta et al., 2010) and Janiak (Janiak et al., 2007) could have been partially mediated *via* GPR55.

In summary, CB1 antagonists may exert beneficial effects directly on the kidney and indirectly through metabolic improvement. It will be particularly important to dissect in more detail the function of other cannabinoid-like receptors in the different kidney areas, especially in the face that GPR55 is expressed in the kidney (Jenkin et al., 2015a; Zhou et al., 2021), and *in vitro* studies have reported its interaction and reciprocal modulation with CB1 and CB2 signalling pathways (Kargl et al., 2012; Balenga et al., 2011).

### 2.2 CB2 in glomerular pathophysiology and CKD

Activation of either CB1 or CB2 can lead to different pathophysiological mechanisms in the kidney. For example, the activation of CB2 can either be detrimental or protective for kidney physiology depending on disease aetiology. CB2 activation is protective of kidney function by maintaining the expression and location of slit diaphragm proteins and reducing albumin excretion in a mouse model of DN (Barutta et al., 2011; Zoja et al., 2016). Moreover, there is conflicting evidence in other models, such as in UUO mice, in which CB2 has been pointed out as a major mediator of kidney fibrosis in tubular cells (Zhou et al., 2021), while other reports suggest a renoprotective effect by reducing inflammatory markers and fibrosis (Swanson et al., 2022; Nettekoven et al., 2016). Regardless of the discrepancy in the role of CB2 in different kidney diseases, the evidence to date suggests that the activation of this receptor is an important potential target for the treatment of DN. In a mouse model of STZ-induced DN, the activation of CB2 was necessary to preserve the slit diaphragm (Barutta et al., 2011). Combined therapy with CB1 blockade proved to be more effective at reducing the expression of inflammatory and fibrotic markers such as TNFα, fibronectin, and collagen type IV, reducing mesangial expansion, monocyte infiltration, and albumin excretion, more than single therapy with AM1241(CB2 agonist) or AM6545 (CB1 antagonist) alone (Barutta et al., 2017). This synergy could be explained by the fact that CB1 blockade preserved slit diaphragm without affecting inflammatory and fibrotic markers, while CB2 activation reduced fibrosis, monocyte infiltration, and slit diaphragm preservation in its own. Therefore, the combined pharmacological approach targeted both the glomeruli directly and the immune system activation that contributes to kidney damage as well. In fact, in CB2 −/− knockout mice, STZ-induced DN caused structural and functional changes in the kidney, evidenced by increased albumin excretion, slit diaphragm loss, monocyte infiltration, and fibrosis, which were larger than those observed in wild-type diabetic mice (Barutta et al., 2014). However, when the bone marrow of CB2 wild-type mice was transplanted in the CB2−/− DN mice, the only CB2 expression observed on the kidney was in the infiltrating monocytes, highlighting that CB2 plays a role in modulating the immune response during DN (Barutta et al., 2014). This is in line with another study using a CB2-GFP reporter system introduced in mice, in which it was observed that either in cisplatin and UUO mice, there was no expression of GFP (CB2) in kidney cells, and only some rare interstitial cells were GFP positive, possibly infiltrated white cells (Boals et al., 2025). It would be interesting to use this reporter system across different kidney disease models to dissect the specific contributions of kidney cells and immune cells to other diseases. Also, Barutta and colleagues did not evaluate the types of inflammatory cells in the kidney using their CB2−/− chimera experiments, so the specific role of CB2 in the immune response to kidney pathology is incomplete. In other models of DN, such as in BTBR *ob/ob* mice, the CB2 agonist HU910 also reduced proteinuria (Zoja et al., 2016). It would be very informative to generate and evaluate the specific deletion of CB2 on different kidney cells, especially considering that the expression of this receptor in the kidney is relatively weak.

In other CKD models, such as a rat model of obesity-induced renal damage, a decrease in CB2 expression has been reported, while its activation improves proteinuria and reduces markers of kidney fibrosis (Jenkin et al., 2015a). It is noteworthy that, in both diabetes and obesity, CB2 is downregulated; however, its activation in these models ameliorates glomerular damage. Conversely, CB1 is upregulated in diabetes and obesity models, despite evidence showing that its blockade improves renal function (Janiak et al., 2007; Barutta et al., 2010). Therefore, these seemingly counterintuitive biological responses may reflect a maladaptive process occurring in podocytes. Indeed, several pathophysiological mechanisms suggest that kidney disease elicits responses in podocytes that are ultimately deleterious or maladaptive to the cells themselves (Nagata, 2016; Yeh et al., 2024; Cunanan et al., 2025; Zhang and Guo, 2025). Collectively, these findings support the notion that dysregulation of the endocannabinoid system contributes to podocyte injury by promoting compensatory signalling pathways that paradoxically may impair podocyte integrity and function.

### 2.3 The role of cannabinoid-like receptors in CKD

The function of GPR55 and other cannabinoid-like receptors in the kidney is scarce. However, agonists and antagonists of GPR55 were used in a model of obesity-induced nephropathy (Simcocks et al., 2019), in which activation of GPR55 (by the O1602 agonist) reduced weight gain and urinary albumin excretion. However, blockade of the receptor (antagonist O1918) also improved albuminuria and kidney inflammation. These compounds may be incompletely characterized, or other cannabinoid receptors may interfere with GPR55 signalling. For example, O1918 is also an antagonist at GPR18 (McHugh et al., 2010), a receptor of unknown function in the kidney. In addition, it has been demonstrated that CB1 can heteromerize with GPR55 and regulate the latter’s activity (Kargl et al., 2012). Similarly, physical interaction and signalling regulation between GPR55 and CB2 have also been reported (Balenga et al., 2014). Thus, CB1/CB2 and GPR55 seem to be functionally related in controlling kidney dynamics.

There is also insufficient information regarding the expression of other cannabinoid-like receptors, such as GPR18, GPR35, and GPR55, in the kidney. For instance, while the expression of GPR55 was increased in obesity-induced nephropathy (Simcocks et al., 2019), no changes were observed in STZ-induced diabetic rats (Jenkin et al., 2015a). These studies did not analyse GPR55 expression in the different kidney compartments, which could have explained the differences between the two studies. Also, the authors did not investigate whether changes in GPR55 expression occurred throughout different stages of the nephropathy models, which could have underestimated the real pathophysiological significance of GPR55, since we do not know how dynamic the regulation of the ECS is in CKD. Regarding GPR18, there is almost no information on its function in the kidney, apart from the fact that the O1918 treatment (antagonist of both GPR18 and GPR55) improved albuminuria despite the upregulation of inflammatory cytokines (Simcocks et al., 2019). In addition, in the UUO model, a model of AKI and CKD, there was a marked upregulation of GPR18 (Lu et al., 2026). To our knowledge, the role of GPR35 has not been studied in the kidney; however, using a Gpr35−/− mouse, it was recently suggested to play a role in the development of hypertension (Divorty et al., 2018), while in the CNS, pharmacological stimulation of hippocampal GPR35 may be anxiogenic (López-Lariz and Marichal Cancino, 2026). Thus, it is plausible that GPR35 could indirectly influence renal processes through central or systemic mechanisms; however, there is currently no direct evidence linking this receptor to activation of the hypothalamic–pituitary–adrenal axis or stress hormone regulation.

Until now, there is almost no information about the expression and function of other orphan receptors phylogenetically related to CB1 and CB2, such as GPR3, GPR6, and GPR12, which are more related to the lysophosphatidic acid receptor family (LPA), and sphingosine-1 phosphate receptors (S1PRs) (Morales and Reggio, 2017). Of those, only GPR3 has been found expressed in the kidney (Iismaa et al., 1994). Thus, given the need for effective therapies for CKD and the pivotal role of cannabinoid receptors CB1 and CB2 in kidney pathophysiology, it would be important to interrogate whether other cannabinoid-like receptors are also involved in CKD.

Altogether, cannabinoid signalling is dysregulated in kidney diseases. Despite promising results in preclinical models of DN or obesity-induced nephropathy, more research is needed to understand the specific roles of the different cannabinoid receptors and their mechanisms of action. One possible role of cannabinoids could be regulating oxidative and nitrosative stress (Udi et al., 2017, Udi et al., 2020; Li et al., 2020). For example, cannabidiol (CBD) reduced nitrotyrosine formation, oxidative stress, and adhesion molecules in endothelial cells exposed to high glucose (Rajesh et al., 2007). *In vivo*, a dual inhibitor of NOS and CB1 also reduced oxidative stress (Udi et al., 2020). Other mechanisms may involve a reduction in fibrosis independent of TGF-β expression (Lecru et al., 2015; Suzuki, Fleig and Penner, 2023) or fibrosis mediated by downstream effectors of cannabinoid receptors, such as β-arrestin and β-catennin (Zhou et al., 2021). In this regard, it is important to notice that synthetic cannabinoids could work as biased agonists towards β-arrestin or Gi branches of the cannabinoid receptor pathway, which could explain discrepancies between studies (Shen et al., 2024; Shen et al., 2025), and this will need to be investigated for their clinical translation, minimizing unwanted side effects. Another challenge will be to understand the systemic effects of cannabinoids, such as the reduction of systolic blood pressure, decrease in body fat, and insulin resistance that could indirectly improve kidney function (Jenkin et al., 2016; Simcocks et al., 2019). Therefore, the precise mechanisms of cannabinoid signalling have not been completely disentangled.

### 2.4 CB1 in tubular pathophysiology

In addition to the direct effects of cannabinoids on glomeruli in CKD models, several studies also highlight dysregulation of the endocannabinoid system in tubules. Notably, the contribution of tubular damage in the progression of CKD is well established (Magri and Fava, 2009; Zeni et al., 2017). Several pathological mechanisms induce tubular toxicity in CKD and AKI. For example, proteinuria/albuminuria increases the endocytosis of proteins from the filtrate, which, if sustained, leads to apoptosis through endoplasmic reticulum stress and caspase 12 activation, both *in vivo* and *in vitro* (Ohse et al., 2006). Protein overload also increases ROS production and inflammatory biomarkers as demonstrated by Morigi and colleagues using the human proximal tubular cell line HK-2 (Morigi et al., 2002) in which H_2_O_2_ produced in the mitochondria was induced by albumin exposure (Morigi et al., 2002; Makhammajanov et al., 2024). It has also been shown that the fatty acids bound to albumin cause tubular toxicity by decreasing mitochondrial membrane potential and triggering peroxide production (Ruggiero et al., 2014). In diabetes, the sustained glucose overload undergone by proximal tubular cells also increases their metabolic demands, ROS production, induces a metabolic shift towards glycolysis, and leads to cell death independently of protein overload (Hinden et al., 2022). In this regard, inhibitors of the sodium-glucose transporter SGLT2, necessary for glucose reabsorption in the proximal tubule, have been shown to be very effective in the treatment of DN progression (Wanner et al., 2016; Perkovic et al., 2019), perhaps by reducing the activation of mTORC1 in proximal tubular cells (Kogot-Levin et al., 2020). Noteworthy, despite the repair mechanism taking place in kidney tubule such as epithelial cell replacement, these are sometimes insufficient for achieving a complete repair, which explains the augmented risk of CKD development in patients who have previously experienced and AKI episode, supporting the maladaptive repair hypothesis of tubular cells (Ishani et al., 2009; Chawla et al., 2011; Ferenbach and Bonventre, 2015).

Multiple lines of evidence highlight the dysregulation of this system specifically within the kidney tubule. For instance, since ECS plays a major role in nutrient sensing and metabolism at the organism and cellular levels, the role of CB1 in proximal tubular cells was investigated by means of its specific deletion in a mouse model of diet-induced obesity (Udi et al., 2017). The authors reported that lipid accumulation in the proximal tubule was reduced upon CB1 deletion, in concert with the activation of AMPK signalling (Udi et al., 2017), a pathway involved in lipid oxidation that prevents tubular lipotoxicity in several models of kidney disease (Rajani, Pastor-Soler and Hallows, 2017; Harley et al., 2022). Also, in a STZ-induced DN model, lipid accumulation, tubular hypertrophy, and fibrosis markers upregulated in hyperglycaemia were markedly decreased by curcumin treatment (Soetikno et al., 2013). Although curcumin has potent free radical scavenger and anti-inflammatory effects, it could also work through CB1 blockade, as it was previously observed both *In vivo* and *In vitro* in a model of hepatic fibrosis in which curcumin decreased extracellular matrix (ECM) protein expression (Zhang et al., 2013). Interestingly, it must be highlighted that curcumin was recently demonstrated to be a GPR55 agonist as well (Harada et al., 2022) a cannabinoid-like receptor implicated in kidney pathology.

In DN models, CB1 blockade downregulates GLUT2, prevents palmitic acid–induced apoptosis (Lim et al., 2010), and reduces fibrosis in tubular cells (Hinden et al., 2018). Consistently, in the UUO model, CB1 mediates fibrosis (Lecru et al., 2015), while in cisplatin-induced nephropathy, its activation promotes oxidative and nitrosative stress as well as tubular apoptosis, effects prevented by the CB1 antagonist cannabidiol (CBD) (Pan et al., 2009). These findings align with the detrimental role of CB1 activation in glomeruli described in DN (Barutta et al., 2010; Jourdan et al., 2014). Translationally, a phase 1 trial with the anti-CB1 antibody GFB-024 demonstrated good tolerability and pharmacokinetics (Bilic et al., 2022), with further studies planned in overweight individuals with T2D. By contrast, a phase 2 trial of the CB1 antagonist INV-022 in DN patients failed to improve outcomes and was associated with neuropsychiatric side effects (Inversago Pharma Inc., 2025; NCT05514548; Cherney et al., 2026). Overall, while experimental evidence consistently supports CB1 as a therapeutic target in CKD, clinical translation remains challenging and highlights the need for more selective strategies.

### 2.5 CB2 in tubular pathophysiology

The pathophysiological role of CB2 receptors in tubular pathophysiology remains controversial, with studies primarily reporting protective actions resulting from CB2 activation, while others have reported detrimental effects, depending on the experimental context. Earlier studies, in models of AKI, reported that CB2 agonism reduced inflammation, apoptosis, microcirculatory dysfunction, and tubular damage, leading to improved renal function (Trojnar et al., 2020; Pressly et al., 2018; Nettekoven et al., 2016; Chafik et al., 2022). Moreover, deficiency of CB2 receptors exacerbates renal injury in cisplatin-induced nephrotoxicity and diabetic nephropathy, suggesting a protective role of endogenous CB2 signalling (Mukhopadhyay et al., 2010; Barutta et al., 2014). Thus, the initial evidence points to the beneficial effects of CB2 activation. Nevertheless, studies from Zhou and colleagues reported deleterious effects *via* CB2 receptors in tubular compartments. In their models, CB2 activation was associated with oxidative stress, lipid accumulation, and fibrosis, while modulation of endocannabinoid metabolism improved renal outcomes (Zhou et al., 2021; Zhou et al., 2024). The same research group showed that in advanced-stage CKD patients, including DN ones, and in UUO mice, there was a higher content of 2-AG, a ubiquitous endocannabinoid frequently linked to the immune system and a CB2 ligand, than in healthy subjects. Also, UUO triggered CB2 upregulation, MAGL (2-AG hydrolysing enzyme) downregulation, tubular lipid accumulation, and fibrosis, which were prevented by MAGL kidney infusion or tubular-specific MAGL knockin (Zhou et al., 2024). In line with these findings, CB2 inhibition has also shown beneficial effects in certain AKI models (Erge et al., 2024). Hence, current evidence indicates that CB2 may exert context-dependent effects in kidney disease.

The above discrepancies could be attributed to several reasons. For instance, in the work of Zhou and colleagues (Zhou et al., 2018), the CB2 −/− mice showed diminished tubular fibrosis, but indirect effects in other cell types caused by CB2 absence cannot be ruled out. Also, CB2−/− could have shifted the “ECS tone” in immune cells, for example, dysregulating physiological CB2 signalling. Finally, the increase in 2-AG reported by Zhou and collaborators could have activated CB1 and GPR55 as well. To support this notion, 2-AG is a full agonist at GPR55 (Ryberg et al., 2007) but just a partial agonist at CB2 (Ben-Shabat, et al., 1998). On the other hand, the authors indicated that in the UUO model, they detected CB1 and CB2 heterodimer formation even though no changes in CB1 expression were observed (Zhou et al., 2021). In this regard, physical interaction and reciprocal regulation between CB1 and CB2 have been observed in the rat brain (Callen et al., 2012), and CB1 is more abundant than CB2 in the kidney (Lecru et al., 2015; Larrinaga et al., 2010). As recently discussed by de Jesus and collaborators (de Jesus et al., 2026), most pharmacological and genetic studies support a renoprotective role of CB2 activation, while discrepant findings are likely explained by methodological and pharmacological differences. These findings underscore the complexity of CB1/CB2 interactions and suggest that the effects of CB2 deficiency in kidney disease models should be interpreted cautiously, as differences in disease type and stage, as well as the use of genetic *versus* pharmacological approaches, are likely important.

### 2.6 GPR55 in tubular pathophysiology

The role of GPR55 and other cannabinoid-like receptors in tubular pathophysiology is scarce. For example, in a mouse model of sepsis-induced AKI, the GPR55 antagonist CID16020046 prevented inflammation and tubular apoptosis (Chen et al., 2022). However, in STZ-induced DN rats, curcumin, a GPR55 agonist, decreased tubular hypertrophy and fibrosis (Soetikno et al., 2013). Nonetheless, as it has been discussed, curcumin could also target other cannabinoid receptors, and therefore, we cannot conclude that the results of Soetikno and collaborators are mediated by GPR55 only. In addition, curcumin is also known to be a potent free radical scavenger, decrease insulin resistance and body fat composition (El-Moselhy et al., 2011), and all these factors play an independent role in kidney disease. It would be interesting to carry out *in vitro* and *in vivo* studies with GPR55 inhibitors and curcumin to investigate the relative contribution of this receptor in tubular pathology and the beneficial effects of curcumin.

It must be highlighted that there is a mutual regulation between cannabinoid receptors (CB1 and CB2) and GPR55, which is still under investigation. However, direct interaction of CB1 and GPR55 has been demonstrated, showing that CB1 receptors block the signalling of GPR55 *in vitro* (Kargl et al., 2012), and the formation of heteromers of CB2 and GPR55 has also been reported (Balenga et al., 2014). Specifically in proximal tubular cells, albumin and high glucose, known stressors of the kidney tubule, caused an upregulation of both *GPR55* and *CB1*, but the mechanisms involved in this upregulation have not been addressed (Jenkin et al., 2015a), nor whether GPR55 is involved in tubular pathology. The most significant effects of ECS signalling activation in kidney disease models are presented in Table 1.

**TABLE 1:** Critical findings in which cannabinoid receptors (CB1 and CB2) and GPR55 have been targeted in AKI and CKD models.

Disease model	Treatment	Findings	References
STZ-induced DN mice	CB1 inhibition (AM251)	↑ Preservation of slit diaphragm protein (Nephrin, Podocin, ZO-1) ↓ Albumin excretion	Barutta et al. (2010)
Prediabetic Zucker diabetic fatty rats (ZDF)	CB1 inhibition (JD5037) for 90 days	↓ Proteinuria, oxidative stress, AngII, and CB1 expression ↑ Preservation of the slit diaphragm	Jourdan et al. (2014)
STZ-induced DN mice	CB2 activation (AM1241)	↑Preservation of slit diaphragm protein (Nephrin, Podocin, ZO-1) ↓ Albumin excretion, kidney/body weight ratio, monocyte infiltration, fibrosis (Tgf-β)	Barutta et al. (2011)
STZ-induced DN mice	Dual CB1 inhibition (AM6545), CB2 activation (AM1241)	↑ Preservation of the slit diaphragm ↓ less mesangial expansion, monocyte infiltration, and fibrosis than single therapy	Barutta et al. (2017)
Uninephrectomised + STZ diabetic rats	CB1 agonist (oleamide), CB1 blockade (AM6545)	CB1 activation = DN + uninephrectomy CB1 inhibition = ↓ Albumin excretion, serum creatinine, BUN, renal hypertrophy, collagen deposition whin	Medapati et al. (2021)
STZ-induced DN mice	Podocyte specific CB1 deletion	↑ GFR ↓ Tubular and glomerular dysfunction	Jourdan et al. (2018)
UUO and cisplatin kidney injury in mice	CBD and CBGA	↑Improved renal function (↓BUN, serum creatinine, albuminuria ↓dilated tubules, inflammatory cytokines (e.g., Tnf-α, Tgf-β, Il6β), tubular apoptosis	Suzuki, Fleig and Penner (2023)
UUO and IRI mice	Selective CB2 agonist	↓40% decrese in tubulointerstitial fibrosis and plasma markers of tubular damage	Nettekoven et al. (2016)
UUO mice	Selective CB2 agonist SMM295 in UUO	↓Tubulointerstitial fibrosis, and DNA damage (^m^γH2AX)	Swanson et al. (2022)
UUO mice	CB1 −/− KO, and rimonabant (CB1 inverse agonist)	↓ Macrophage infiltration, mesangial expansion, fibrosis	Lecru et al. (2015)
UUO and IRI mice	MAGL knockin proximal tubular cells, 2-AG kidney infusion	↓ MAGL knockin ↓ proximal tubular cell lipid accumulation, fibrosis, and β-catenin activation	Zhou et al. (2024)
UUO mice, IRI, folic acid-induced nephropathy	CB2 inverse agonist (XL-001), CB2 overexpression, β-arrestin knockdown	CB2 blockade or β-arrestin ↓ β-catenin nuclear translocation, fibrosis CB2 forced expression ↑ matrix proteases, α-SMA, β-catenin	Zhou et al. (2021)
UUO, IRI and adriamycin kidney injury	CB2 −/− KO mice, CB2 inverse agonist (XL-001)	↑ CB2, β-catenin, tubular hypertrophy, fibrosis in nephropathy models ↓ fibrotic area by CB2 KO or XL-001	Zhou et al. (2018)
Obese *fa/fa* Zucker rats	Rimonabant (CB1 inverse agonist)	↓ plasma triglycerides, cholesterol, fatty acids, glucose, norepinephrine ↑ Creatinine clearance, improved renal structure, and function	Janiak et al. (2007)
High-fat diet-induced obesity in rats	CB1 antagonist (AM251)	↓ Weight gain, leptin, kidney weight, plasma creatinine, albumin excretion, tubular diameter ↑ Creatinine clearance	Jenkin, O’Keefe, et al. (2015b)
High fat diet-induced obesity in rats	CB2 agonist (AM1241), antagonist (AM630)	↓ Systolic blood pressure, plasma leptin and kidney weight, tubular diameter upon CB2 activation ↑ Albumin excretion, kidney weight in CB2 antagonism (AM630)	Jenkin et al. (2016)
Glycerol-induced AKI rats	CB1 antagonist (AM251) CB2 antagonist (SR144528) CB2 and CB1 agonist (WIN55212-2)	↓ Serum creatinine, renal damage score upon CB2 blockade, and CB1 activity ↑ Urine volume, creatinine clearance upon	Erge et al. (2024)
High-fat diet-induced obesity in rats	GPR55 agonist (O-1602) and antagonist (O-1918)	↓ Adiposity, Ghrelin, and Leptin in O1602 ↑ lean mass, inflammatory cytokines by O1918 treatment ↑ kidney weight, and liver damage with both drugs	Simcocks et al. (2019)
Sepsis-induced AKI in mice	GPR55 antagonist (CID16020046)	↓ Plasma creatinine, BUN, NAGL, KIM-1, inflammatory markers, and apoptosis	Chen et al. (2022)
STZ-induced DN rats	CB2 activator (β-caryophyllene)	↓ Plasma glucose, creatinine, BUN, kidney weight, urine volume ↑ Body weight, creatinine excretion, GSH	Kumawat and Kaur (2024)
High-fat diet-induced obesity in mice	Proximal tubular cell-specific CB1 −/−	↓ albumin/creatinine ratio, inflammation, fibrosis, tubular lipid accumulation ↑ AMPK activation, lipid oxidation	Udi et al. (2017)

## 3 The ECS in the risk factors for CKD

### 3.1 The ECS in glucose control

It's worth noting that targeting the ECS can not only directly affect kidney function, but it can also indirectly impact hemodynamic, glycemia, dyslipidaemia, obesity and systemic inflammation. All factors that contribute to the progression of CKD (Arceri et al., 2023). For example, it must be remembered that approximately 30% of CKD cases can be attributed to diabetes, which is mostly due to obesity; likewise, another third of CKD cases are due to high blood pressure (Deng et al., 2025). Therefore, it is likely that the positive effects of cannabinoids in CKD models go beyond the direct effects on the kidney.

Cannabinoid activity, particularly *via* CB1 receptors, exerts a multifaceted influence on glucose homeostasis. For example, in skeletal muscle, blocking CB1 receptors enhances glucose uptake by means of GLUT4 membrane translocation, and this effect is decreased in muscle from insulin-resistant obese rats (Lindborg et al., 2010). On the contrary, on visceral adipose tissue, it is CB1 activation, and possibly CB2 also, that causes an increase in GLUT4-mediated glucose uptake (Pagano et al., 2007). In addition, epidemiological studies have shown a decrease in fasting insulin and glucose levels between marijuana users compared to non-users (Penner, Buettner, and Mittleman, 2013; Di Marzo and Silvestri, 2019). Although these findings are conflicting with the view of CB1 activation-induced insulin resistance, it must be remembered that marijuana contains a large number of cannabinoids, which can antagonize the actions of CB1, such as CBD, and can also activate a large number of receptors involved in reducing adiposity and controlling glucose metabolism (De Petrocellis et al., 2011; Rodríguez-Carreiro et al., 2025). Thus, the most accepted view is that CB1 blockage is an effective strategy for the treatment of obesity, diabetes, and metabolic syndrome.

In fact, rimonabant, a CB1 blocker, resulted in very effective modulation of glucose and lipid metabolism centrally and peripherally, but was later removed due to the potential increase of unwanted depressive symptoms (Sam, Salem and Ghatei, 2011). However, peripherally restricted CB1 receptor antagonists (e.g., JD5037, AM6545) have also demonstrated promising results in rodent models of obesity and diabetes without central nervous system effects (Moreno, Cavic and Canela, 2021), and hopefully these results will be translated to the clinical setting.

### 3.2 The ECS in obesity and lipid metabolism

In animal and *in vitro* studies, CB1 inhibition has been shown to disrupt orexigenic behaviour and induce lipolysis, whereas its activation induces lipogenesis in liver and adipose tissue, by means of fatty acid synthase (FAS) and SREBP-1c transcription (Cota et al., 2003; Osei-Hyiaman et al., 2005). Another mechanism responsible for the increase in lipid oxidation upon CB1 blockade involves increased expression of CPT1 in adipose tissue and skeletal muscle (Wei et al., 2018). These metabolic changes are intertwined with renal function. For example, in Zucker obese rats, CB1 inhibition not only improved renal function but also decreased plasma leptin, triglycerides, and cholesterol (Janiak et al., 2007). Metabolic changes such as a decrease in body weight have also been observed upon GPR55 activation (Simcocks et al., 2019). In addition, the CB2 agonist AM1241 not only reduced markers of kidney fibrosis and urinary protein excretion but also had a positive effect in reducing weight gain, decreasing plasma leptin and systolic blood pressure in obese rats (Jenkin et al., 2016), which stresses how cannabinoids could target systems involved in CKD progression.

Regarding epidemiological data, chronic cannabis users tend to have a lower prevalence of metabolic syndrome and obesity. For example, in a correlation analysis of the “National Health and Nutrition Examination Survey” (NHANES) data, in more than 8,000 adults, the prevalence of metabolic syndrome among marijuana users was 13.8% as compared to 19.5% in non-users (Vidot et al., 2016). These data have also been corroborated in prospective studies, one of which included 33,000 adults, that showed marijuana use correlated with a negative trend in BMI as compared to the increase in BMI of non-users (Alshaarawy and Anthony, 2019). CBD, one of the main components of cannabis has shown benefits in lipid modulation; in rodent models, CBD interact with receptors and systems such as GPR55, TRVP, PPAR between others and help to decrease sphingolipid levels across adipose tissue, muscle, and brain, restoring insulin sensitivity and mitigating insulin resistance, which suggests potential roles in the management of dyslipidaemia and metabolic syndrome (Moreno, Cavic and Canela, 2021). Another possibility to explain epidemiological results of marijuana users is the interaction of cannabinoids and the host microbiota. For instance, in the DIO mouse model, THC administration prevented alteration in gut microbiota observed in obesity, such as the increase in the Firmicutes/Bacteroidetes ratio, as well as increasing Akkermansia muciniphila upon THC treatment (Cluny et al., 2015). Interestingly, these changes were not observed in lean mice, highlighting the context-specific regulation of cannabinoids and microbiota. Finally, it is also possible that chronic use of cannabinoids decreases “CB1 sensitivity”. For instance, using a specific CB1 ligand for positron emission tomography (PET), it was shown that chronic cannabis use decreased receptor availability in several brain areas (Ceccarini et al., 2015). Thus, the endocannabinoid system regulation is highly complex, and how this system regulates metabolism and complications, such as kidney diseases, requires further dissection (Di Marzo and Silvestri, 2019).

In summary, the evidence so far highlights that CB1 promotes: energy storage, hyperglycaemia, lipogenesis, inhibits fatty acid oxidation and adiponectin secretion, elevates triglyceride and cholesterol levels, pancreatic β-cell apoptosis, oxidative stress, and inflammation, forming a pathogenic cycle that can culminate in type 2 diabetes and its associated microvascular complications that leads to renal injury (Simankowicz and Stępniewska, 2025; Moreno, Cavic and Canela, 2021; Dörnyei et al., 2023).

### 3.3 The modulation of inflammation by the ECS

Endocannabinoids such as AEA, 2-AG and phytocannabinoids like CBD, THC exert potent anti-inflammatory effects, regulated through CB1 and CB2 as well as non-canonical pathways (e.g., adenosine A2A receptor inhibition across CBD) (Lowe et al., 2021). For example, CBD has been shown to regulate the immune response in NOD mice, a model of immune-induced diabetes, by decreasing IFN-γ and TNF-α, for example, which ultimately decreases pancreatic leucocyte infiltration and diabetes (Weiss et al., 2006). Moreover, CB2 agonists such as JWH-133 reduce body weight, insulin resistance, and inflammatory M1 macrophages through activation of the Nrf2 pathway (Wu et al., 2020). Along the same line, CB2 activation by JWH015 decreased body fat, reduced adipocyte size, and decreased inflammatory cytokines (Verty et al., 2015). However, JWH015 can also activate GPR55, which could also improve metabolic outcomes, since GPR55 −/− null mice have excess lipid tissue, decreased activity, as well as inefficient insulin secretion (Meadows et al., 2016; Liu, Ruz-Maldonado, and Persaud, 2024). Similarly, in human patients, variants of CB2 with decreased activity (Q63R) were associated with higher BMI, and stem cell-derived adipocytes from obese subjects expressed higher inflammatory cytokines as compared to adipocytes of lean subjects (Rossi et al., 2016; Wu et al., 2020). In addition, in DN models, CB2 activation *via* beta-caryophyllene (BCP), combined with L-arginine, significantly reduces hyperglycaemia, inflammatory cytokines (e.g., IL-6, TNF-α), NF-κB expression, oxidative stress, fibrosis, and collagen deposition in kidney tissue (Kumawat and Kaur, 2024).

### 3.4 The ECS and cardiovascular regulation

The endocannabinoid system influences cardiovascular function through central and peripheral mechanisms. In hypertensive animal models, augmenting endocannabinoid levels led to blood pressure reductions (Dörnyei et al., 2023). For example, in several hypertensive rat models, increases in anandamide and endothelial CB1 expression have been observed compared to normotensive animals, and inhibition of FAAH and CB1 agonists caused an acute hypotensive effect, while CB1 antagonists augmented AngII-induced hypertension (Bátkai et al., 2004). In *ex vivo* microangiometry of different arterioles, AngII-induced vasoconstriction triggered a vasodilator response mediated by CB1 activation, and therefore, in explants from CB1 −/− KO mice, the vasoconstriction of AngII was even higher (Szekeres et al., 2012, 2015). Moreover, CB1 produces sympathoinhibition, TRPV1 may release the vasorelaxant calcitonin gene-related peptide, and GPR55 exerts vasorelaxant actions at the endothelial level (Marichal-Cancino et al., 2013, 2020). Along the same line, 2-AG also produced a hypotensive effect and bradycardia in anesthetized mice mediated by CB1 receptor (Járai et al., 2000). Therefore, CB1 expression forms part of a negative feedback loop taking place in arterioles to counteract blood pressure increases. However, care should be taken before we can translate these findings into the human setting, due to contradictory evidence and incomplete understanding of the many mechanisms involved in blood pressure control. For example, it has been shown that high doses of anandamide increase blood pressure and kidney cortical fibrosis in Dahl salt-sensitive hypertensive rats induced with a high salt diet (Golosova et al., 2022), and in humans, there are mixed results. On the one hand, acute THC administration has been reported to decrease blood pressure, yet chronic use may be associated with elevated prevalence of hypertension (Dörnyei et al., 2023; Vallée, 2023). For example, in epidemiological studies, cannabis and tobacco smoking showed additive effects on subclinical atherosclerosis assessment (Auer et al., 2018). Also, in a recent cross-sectional study, cannabis users showed signs of arterial stiffness, and their serum was less effective at inducing NO *In vitro* compared to non-users’serum (Mohammadi et al., 2025). Therefore, cannabinoids could impact the cardiovascular and metabolic system in opposite directions when targeting kidney disease, for example, decreasing adiposity and insulin resistance in one hand, but inducing hypertension on the other, highlighting the complex role of ECS.

CB1 antagonism has also shown benefits in cardiac function in post-myocardial infarction and improves metabolic profiles (Dörnyei et al., 2023). In a uremic cardiomyopathy model induced by 5/6 nephrectomy, CB1 blockade decreased myocardial fibrosis and ventricular hypertrophy (Lin et al., 2015), and similarly in rats with coronary ligation, rimonabant decreased TGF-β and fibrosis (Slavic et al., 2013). On the contrary, CB2 seems to counteract CB1 in cardiac remodelling, for instance, CB2 −/− KO animals are unable to recover from heart ischemia reperfusion injury, with augmented apoptosis and fibrosis not observed in wild type mice (Duerr et al., 2014). The understanding of the regulation of ECS in the cardiovascular system is still incomplete, but its pleiotropic actions are a promising field for developing therapeutic interventions.

In summary, the combined impact of glycemia regulation, lipid homeostasis, body weight, blood pressure control, and modulation of inflammation directly shapes renal hemodynamic, glomerular filtration, and susceptibility to fibrosis, whereby hyperglycaemia and dyslipidaemia drive glomerular hypertrophy, mesangial expansion, and podocyte loss.

## 4 Future perspectives and open questions

**FIGURE 1:**
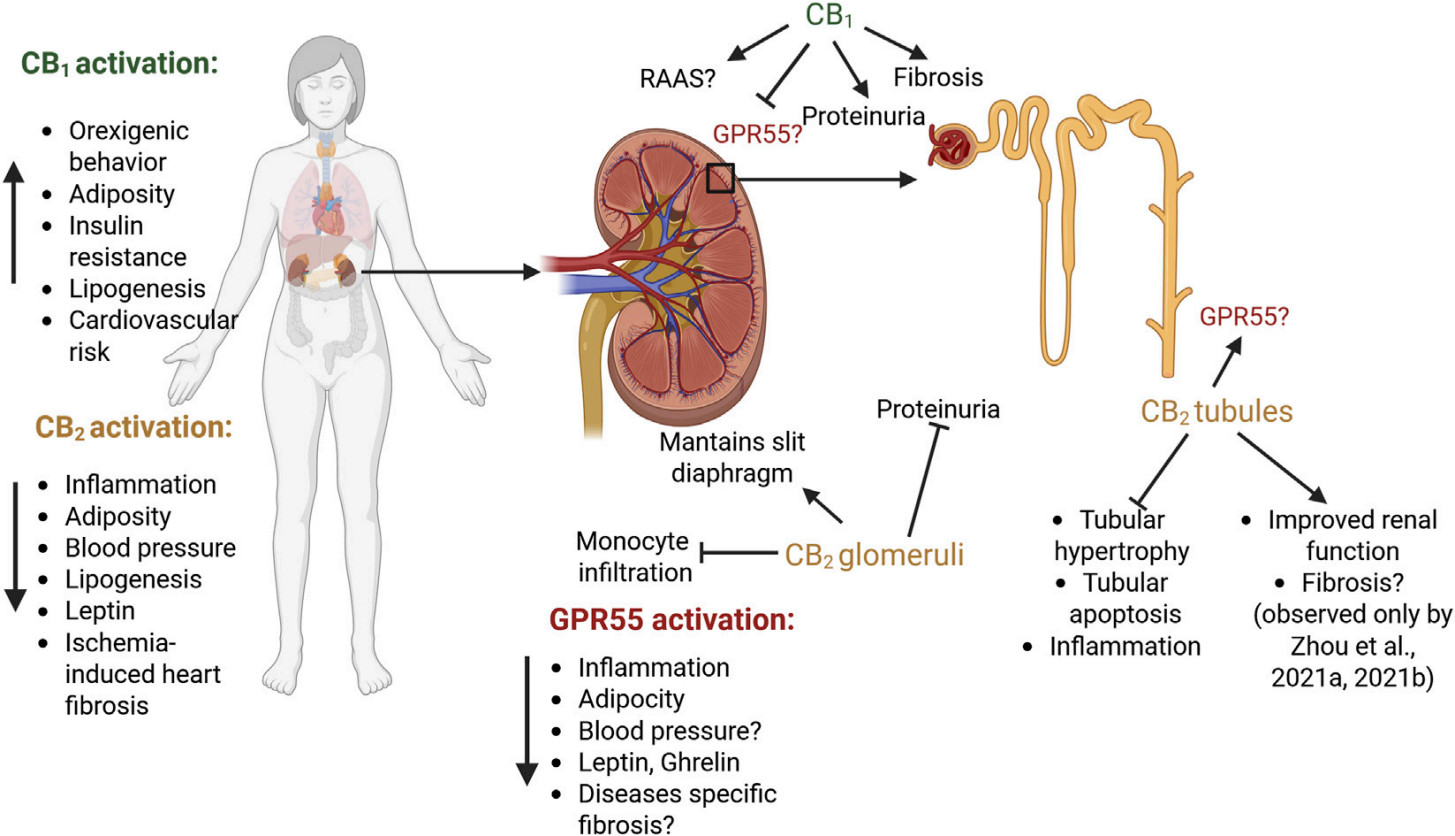
Cannabinoid receptor signalling in renal injury and repair: established, emerging, and unresolved mechanisms. The image summarizes the established roles of CB1 and CB2 activation in the kidney, along with their key effects on metabolism, inflammation, and cardiovascular function in the context of renal pathophysiology. Question marks indicate areas of uncertainty, including contradictory findings, disease-specific responses, and limited evidence in kidney disorders—particularly regarding the role of GPR55. The image was created with BioRender.com.

In the past sections, we have discussed the many functions of ECS and the use of synthetic cannabinoids for models of CKD and AKI pathologies, with remarkable preclinical results. We have also pointed out the difficulties when comparing studies, taking into consideration: disease aetiology, promiscuity of ligands with various cannabinoid receptors, and the types of outcomes analysed (e.g., fibrosis, albuminuria, hyperglycaemia, blood pressure, nutrient sensing, etc.). In addition, the conflicting evidence in some studies was emphasized, and tentative explanations were provided. In the face of the results, we think that the future of the cannabinoid field in kidney disease is promising and encourages further animal and *in vitro* research aiming to dissect the expression of cannabinoid receptors in longitudinal studies, and to consider the involvement of the so-called orphan receptors, which are functionally related to ECS regulation. Finally, multi-organ analysis is also encouraged, since cannabinoids will ultimately alter the activity of several systems related to kidney pathophysiology, or involved in CKD co-morbidities. Figure 1 schematizes the function of the ECS, emphasizing the kidney physiology.
